# Identification of Angiotensin I-Converting Enzyme Inhibitory Peptides Derived from Enzymatic Hydrolysates of Razor Clam *Sinonovacula constricta*

**DOI:** 10.3390/md14060110

**Published:** 2016-06-03

**Authors:** Yun Li, Faizan A. Sadiq, Li Fu, Hui Zhu, Minghua Zhong, Muhammad Sohail

**Affiliations:** 1 School of Life Sciences and Food Technology, Hanshan Normal University, Chaozhou 521041, China; fl1990@163.com (L.F.); gdzhuhui@126.com (H.Z.); 2 College of Biosystems Engineering and Food Science, Zhejiang University, Hangzhou 310058, China; faizan_nri@yahoo.co.uk; 3 School of Chemistry and Environmental Engineering, Hanshan Normal University, Chaozhou 521041, China; zhongmh@hstc.edu.cn; 4 National Institute of Food Science & Technology, University of Agriculture, Faisalabad 38040, Pakistan; Sohail.nifsat@gmail.com

**Keywords:** ACE-inhibitory peptides, razor clam, enzymatic hydrolysis, *Actinomucor elegans* proteases, identification, MALDI/TOF-TOF MS/MS

## Abstract

Angiotensin I-converting enzyme (ACE) inhibitory activity of razor clam hydrolysates produced using five proteases, namely, pepsin, trypsin, alcalase, flavourzyme and proteases from *Actinomucor elegans* T3 was investigated. Flavourzyme hydrolysate showed the highest level of degree of hydrolysis (DH) (45.87%) followed by *A. elegans* T3 proteases hydrolysate (37.84%) and alcalase (30.55%). The *A. elegans* T3 proteases was observed to be more effective in generating small peptides with ACE-inhibitory activity. The 3 kDa membrane permeate of *A. elegans* T3 proteases hydrolysate showed the highest ACE-inhibitory activity with an IC_50_ of 0.79 mg/mL. After chromatographic separation by Sephadex G-15 gel filtration and reverse phase-high performance liquid chromatography, the potent fraction was subjected to MALDI/TOF-TOF MS/MS for identification. A novel ACE-inhibitory peptide (VQY) was identified exhibiting an IC_50_ of 9.8 μM. The inhibitory kinetics investigation by Lineweaver-Burk plots demonstrated that the peptide acts as a competitive ACE inhibitor. The razor clam hydrolysate obtained by *A. elegans* T3 proteases could serve as a source of functional peptides with ACE-inhibitory activity for physiological benefits.

## 1. Introduction

Hypertension is one of the major global health issues, owing to its chronic nature, wide prevalence and linkage with increased mortality and morbidity which affects approximately 16%–37% of the global population [1]. Long term hypertension is one of the major risk factors and clinical manifestations of arteriosclerosis, cardiovascular diseases, strokes, heart failures, and chronic renal diseases [2,3]. Angiotensin-converting enzyme (ACE, EC 3.4.15.1) is a key enzyme of renin-angiotensin system (RAS) which is known as a cascade that controls the regulation of arterial blood pressure and cardiac output. Angiotensin I is a ten-amino acid peptide produced by the action of rennin on angiotensinogen. Once angiotensin I is formed, it is converted to angiotensin II through the removal of two *C*-terminal residues (His-Leu) by the action of ACE, thus resulting in vasoconstriction, ultimately leading to the increase in blood pressure [4]. In addition, ACE is also known to catalyze the degradation of the vasodilator bradykinin into inactive fragments, which leads to the decrease in vasodilation [5]. Thus, the inhibition of ACE is considered as an effective strategy in designing pharmaceutical drugs for the treatment of hypertension. Synthetic drugs targeting inhibition of ACE are normally used for the clinical treatment of hypertension such as captopril, enalapril, and alcacepril. However, therapies with these drugs are believed to cause side effects including dry cough, renal failure, skin rashes, and angioneurotic edema [6]. So, there is a dire need to find natural ACE inhibitors with lower or no side effect in order to development pharmaceuticals and nutraceuticals for the prevention and remedy of hypertension.

Food protein-derived bioactive peptides are naturally physiologically active peptide fragments encrypted within the sequence of food proteins, and can be released through enzymatic hydrolysis and microbial fermentation. Besides providing adequate nutrients, food protein-derived bioactive peptides possess beneficial pharmacological properties such as antihypertensive, antioxidant, antiproliferative, and immunomodulatory activities [7]. There is great interest among researchers to unreveal food based bioactive peptides which are encrypted within food proteins, with a view to develop functional foods and nutraceuticals. Compared with chemosynthetic drugs, bioactive peptides of food origin are usually considered safe, effective and economical and thus these are healthier and more natural alternative to synthetic drugs [8]. Since the discovery of first ACE-inhibitory peptides from snake venome [9], many ACE-inhibitory peptides have been reported from the protein hydrolysates of foods [10].

Marine fishes, due to phenomenal biodiversity of their habitat and broad spectra of bioactivities, are relatively untapped and rich sources of proteins of high biological value as compared to land animals [5]. Thus, fish and sea food are excellent sources of proteins and can be utilized as an ideal starting material for the production of novel ACE-inhibitory peptides. Enzymatic hydrolysis is a widely used method to release ACE-inhibitory peptides from marine fish proteins. The effectiveness of using this method to generate specific peptide fragments with inhibitory activity mainly depends on the proteolytic enzyme used, hydrolysis conditions and the degree of hydrolysis (DH) achieved. A variety of enzymes including commercial proteases and proteases of microbial origin have been reported for the production of ACE-inhibitory peptides from various marine fish proteins. In particular, a number of novel ACE-inhibitory peptides with good activity have been reported from the enzymatic hydrolysate of shellfish such as oyster [11,12], shrimp [13], hard clam [14] and cuttlefish muscle [15].

Razor clam (*Sinonovacula constricta*) is one of the four major economically cultivated shellfish in China, which has been cultured for hundreds of years [16]. Due to its high nutritional and economical values, razor clam is a popular shellfish food and has been widely cultivated along east coast of China. According to 2015 Fisheries Statistical Yearbook of China (2015), the cultured razor clam yield was more than 786,000 tons in 2014. To date, there is no study aiming to investigate the potential of razor clam to generate ACE-inhibitory peptides which could be exploited as antihypertensive agents in functional foods and nutraceuticals. Therefore, the objectives of this work are two folds: first, to evaluate the ACE-inhibitory activity of the hydrolysates produced with different proteases. Secondly, to purify and identify the potential ACE-inhibitory peptides from the hydrolysate. Furthermore, the inhibitory kinetics of the identified peptide based on Lineweaver-Burk plots were also studied.

## 2. Results and Discussion

### 2.1. Production of Enzymatic Hydrolysates

#### 2.1.1. Proximate Composition of Razor Clam

The results of the proximate composition of razor clam are shown in Table 1. The average values for moisture, protein, fat, carbohydrate and ash are 80.32, 13.68, 1.89, 2.13 and 1.93 g/100 g (fresh weight), respectively. On a dry weight basis, protein was the predominant proximate composition, occupying 69.51% of the dry weight. The protein content of razor clam determined in the present study was higher than reported values for protein (9.09–12.75 g/100 g fresh weight) in Asian hard clam (*Meretrix lusoria*) [17], Veneridae clams (9.00–12.51 g/100 g fresh weight) [18] and surf clam (*Mactra violacea*) (11.9 g/100 g fresh weight) [19]. The value of fat content was consistent with previously reported values for fat content in surf clam (1 g/100 g fresh weight) and Veneridae clams (1.32–2.4 g/100 g fresh weight). Similarly, the reported carbohydrate content in the current study is in the range of carbohydrate value that was previously reported in Veneridae clams (1.72–3.61 g/100 g fresh weight). However, a comparatively higher value for fat content has previously been reported for Asian hard clam (1.58–6.58 g/100 g fresh weight). The results of proximate analysis indicate that razor clam is a rich source of nutrients, particularly protein content, and can be used to produce bioactive peptides.

#### 2.1.2. Degree of Hydrolysis and ACE-Inhibitory Activity of Hydrolysates by Different Proteases

**Figure 1:**
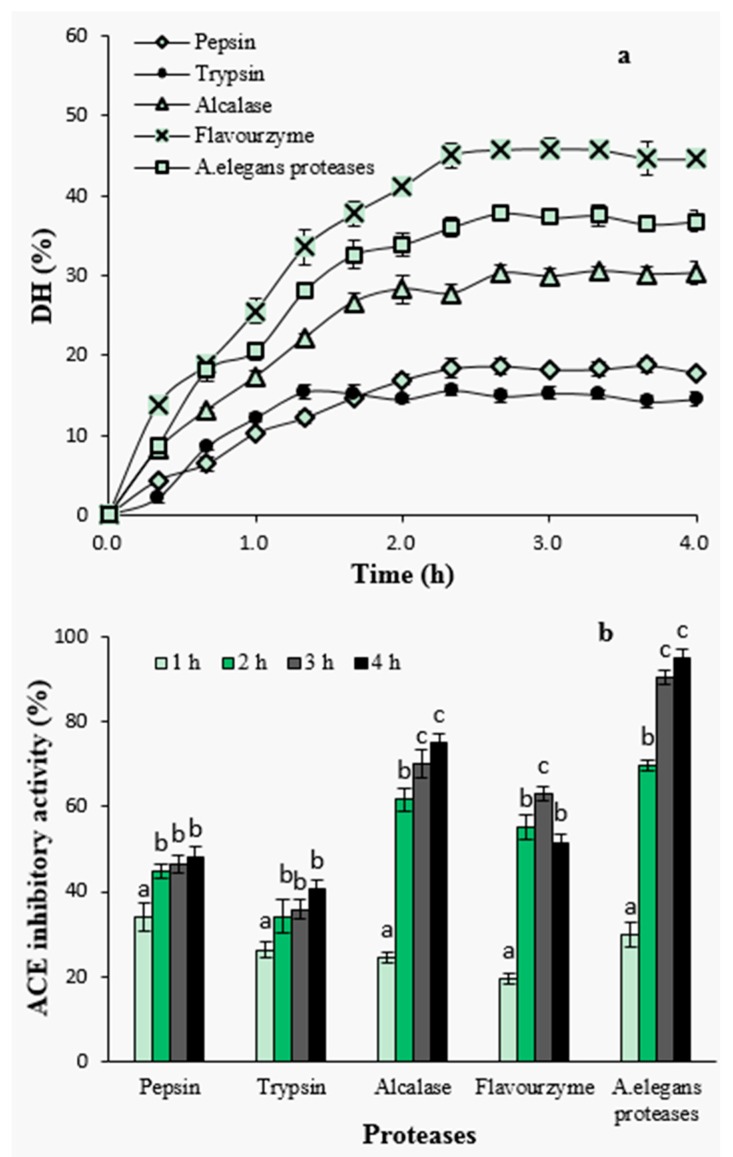
Degree of hydrolysis with proteases during hydrolysis (**a**) and effect of hydrolysis time on angiotensin I-converting enzyme (ACE)-inhibitory activity of hydrolysates (**b**). Different letters indicate significant differences in the same group (*p* < 0.05).

Enzymatic hydrolysis was performed using pepsin, trypsin, alcalase, flavourzyme and crude proteases from *A. elegans* T3. Hydrolysis efficiency was evaluated by measuring degree of hydrolysis (DH) in the hydrolysates that had been generated by using five different proteases (Figure 1a). Overall, the hydrolysis of the razor clam proteins was characterized by a high rate of hydrolysis during the initial 1–2 h; 1 h for pepsin and trypsin hydrolysis, and within 2 h for alcalase, flavourzyme and crude proteases from *A. elegans* T3. The rapid increase in DH indicates that a large amount of peptides were cleaved from proteins and released into hydrolysates at the initial stage. After that, the hydrolysis entered into stationary phase where no apparent increase in DH was observed (Figure 1a). These results represent similar hydrolysis curves that are previously reported for the protein hydrolysates of sardinelle (*Sardinella aurita*) by-products [20], sole and squid [21], yellow stripe trevally (*Selaroides leptolepis*) [22] and catfish (*Pangasius sutchi*) [23]. The rate of enzymatic cleavage of peptide bonds is an important factor determining the rate of DH [24]. During the initial phase of the reaction kinetics, the reaction speed is very fast and thus peptide bonds are easily cleaved resulting in a large number of soluble peptides in the reaction mixture. These peptides also act as effective substrate competitors to undigested or partially digested compact proteins in substrate [25]. Decreased hydrolysis reaction rate during the stationary phase can also be attributed to the limited availability of the substrate, as it is known that the substrate decreases by the reaction time. Also, decrease in enzymatic activity or partial enzymatic inactivation by the time is an important reason of slower degree of hydrolysis during the later stages of the reaction [26].

Among the proteases investigated, hydrolysis with flavourzyme showed higher level of DH during the whole process, reaching a maximum level of 45.87% after 3 h, followed by *A. elegans* T3 proteases (37.84%) and alcalase (30.55%), whereas the lower DH values were observed with pepsin (18.72%) and trypsin (15.67%). The efficiency of proteases in catalyzing the hydrolysis depends on the nature of the substrate proteins and the specificity of proteases towards these proteins. Lower DH value obtained upon tryptic hydrolysis is probably due to trypsin’s specificity, as it is known that trypsin preferentially catalyzes polypeptides on the carboxyl side of basic amino acids (arginine or lysine). In case of pepsin, the enzyme exhibits preferential cleavage for hydrophobic residues, preferably cleaves aromatic residues. However, pepsin is unable to hydrolyse the proline peptide bond efficiently [27]. This may cause resistance to hydrolysis when using pepsin to digest protein substrate containing high content of proline. Similar inefficiency of pepsin has previously been reported when the lowest DH was observed in the pepsin hydrolysate among all the proteases used for barley hordein proteolysis [28].

To investigate the effect of hydrolysis time on ACE-inhibitory activity, samples were taken from the hydrolysates at different time intervals and subjected to ACE-inhibitory activity assay at a concentration of 2 mg peptide/mL (Figure 1b). Among all hydrolysates, the ACE-inhibitory activity increased with increasing hydrolysis time except for flavourzyme-generated hydrolysates. The highest ACE inhibition at a level of 94.79% was observed for the hydrolysates of *A. elegans* T3 proteases after 4 h of hydrolysis. In particular, ACE-inhibitory activity significantly increased during the first stage of hydrolysis which depicts a fast increase in DH at the beginning and its positive influence on the generation of ACE-inhibitory peptides (*p* < 0.05). DH was defined as the percent ration between the fraction of peptide bonds cleaved to the total number of peptide bonds [29], and it has been widely used to evaluate hydrolytic progress. The positive correlation between DH value and ACE-inhibitory activity has been reported in studies on the proteolysis of canola meal [30], cuttlefish muscle [15], palm kernel cake [31] and bovine collagen [32] proteins. It has been suggested that reaching a certain level of DH was contributive to release more active peptides from protein precursors [30]. In the present study, the results of hydrolysis using pepsin, trypsin, alcalase and *A. elegans* T3 proteases were in agreement with these studies. The hydrolysate as a result of *A. elegans* T3 proteases, having higher DH values, showed better ACE-inhibitory activity as well. However, the similar observation was not found in the case of treatment with flavourzyme hydrolysate, which, despite having the highest DH value, showed lower inhibitory activity. Flavourzyme is a complex of protease and peptidases having endoprotease as well as exopeptidase activities. It has been applied to prepare short chain peptides [28] and lower bitter taste of hydrolysates [33]. Action of peptidases can promote the production of peptides of small molecular weight. On the other hand, using this enzyme may also cause degradation of active peptides into shorter inactive peptides or amino acids. Similar inefficiency of using flavourzyme in the production of ACE-inhibitory peptides was reported for red scorpion fish proteins [34].

#### 2.1.3. Peptide Content and ACE-Inhibitory Activity of Ultra-Filtration Fractions

**Figure 2:**
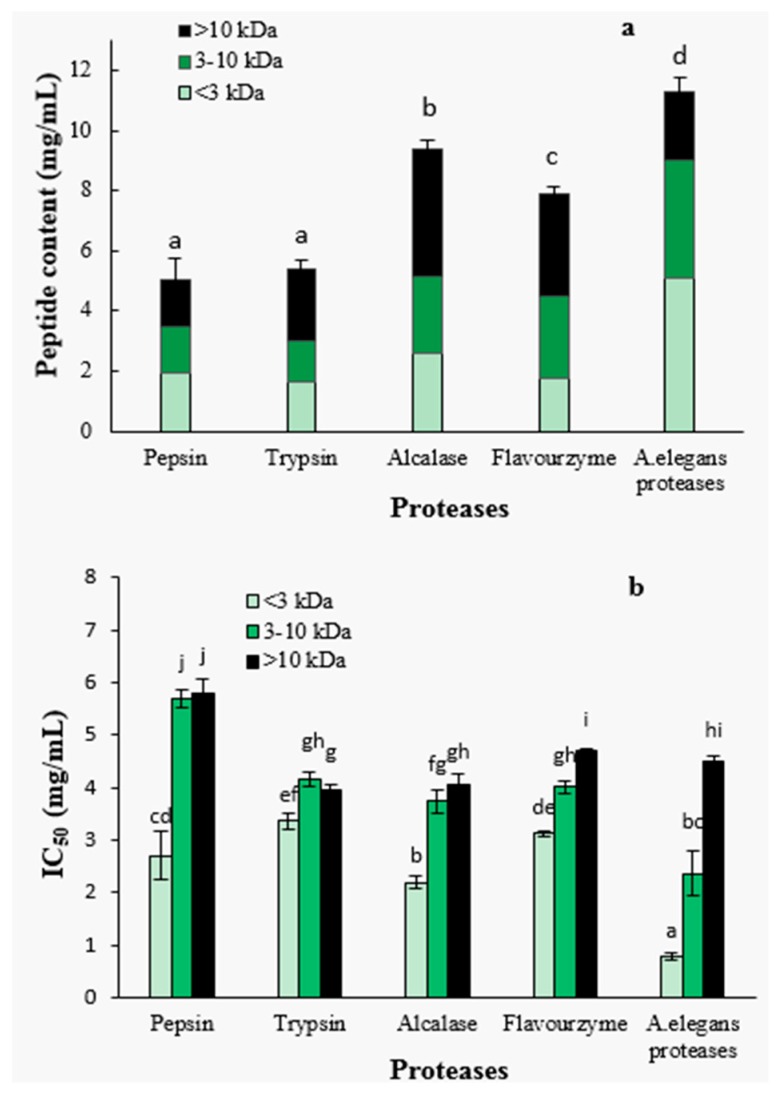
Peptide content (**a**) and IC_50_ value (**b**) of fractions from hydrolysates separated by ultra-filtration. Different letters indicate the mean values are significantly different (*p* < 0.05).

After 4 h of hydrolysis, the hydrolysates obtained with different proteases were further separated by ultra-filtration into three molecular weight fractions, <3 kDa, 3–10 kDa and >10 kDa. The peptide contents and the molecular weight distributions are shown in Figure 2a. The peptide contents of *A. elegans* T3 proteases hydrolysate was significantly higher than that of other hydrolysates (*p* < 0.05), indicating that more peptides were released from protein precursors. Furthermore, *A. elegans* proteases hydrolysate contained larger proportion of the peptides with size below 3 kDa (45.0%) as compared to the other hydrolysates. These results suggest that *A. elegans* T3 proteases is more effective in generating peptides of low molecular weight from razor clam proteins. For flavourzyme hydrolysis, the higher DH did not lead to the higher content of peptides. This can be explained by the fact that flavourzyme contain exopeptidases which release more free amino acids. So the DH value for this enzyme hydrolysate correlates with the content of free amino acids and not with the content of peptides.

The ACE-inhibitory activity was found to be significantly dependent on peptide fraction molecular weight (Figure 2b). The <3 kDa peptide fraction showed significantly higher ACE-inhibitory activity than those of higher molecular weight fractions (3–10 kDa and >10 kDa) for each protease hydrolysate (*p* < 0.05). Specifically, the 3–10 kDa fractions from flavourzyme and *A. elegans* proteases hydrolysates had significantly (*p* < 0.05) higher ACE-inhibitory properties in comparison with >10 kDa fractions. Pepsin, trypsin and alcalase hydrolysates, on the contrary, showed no significant difference in the activity of fractions (3–10 kDa and >10 kDa). The highest ACE-inhibitory activity (lowest IC_50_ value) was found in the <3 kDa fraction of *A. elegans* T3 proteases hydrolysate, with an IC_50_ value of 0.79 mg/mL. Molecular weight is an important determinant for the ACE-inhibitory activity of peptides. It was reported that food protein derived ACE-inhibitory peptides are in the molecular weight range of below 3 kDa [35]. The weak inhibitory activity of high MW peptides are primarily due to the inability of the ACE-catalytic site to bind large molecules [36]. Therefore, based on these result, the <3 kDa fraction of *A. elegans* T3 proteases hydrolysate was used for further purification and identification of active peptides.

### 2.2. Identification of ACE-Inhibitory Peptides

#### 2.2.1. Isolation and Purification of ACE-Inhibitory Peptides

**Figure 3:**
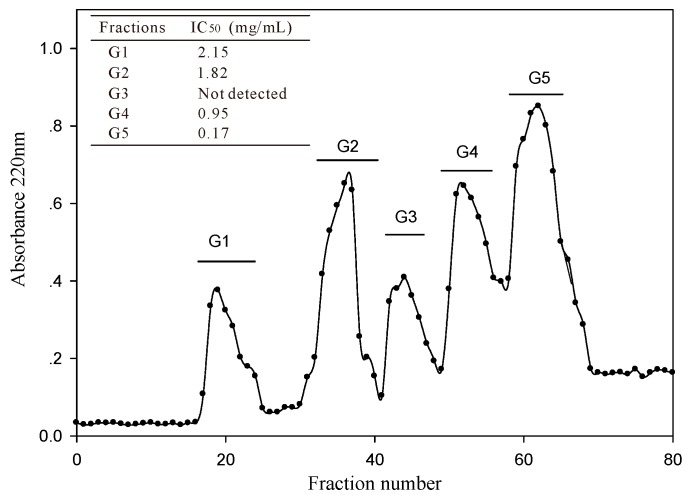
Gel filtration chromatography profile of <3 kDa fraction of *A. elegans* T3 proteases hydrolysate on Sephadex G-15 column.

**Figure 4:**
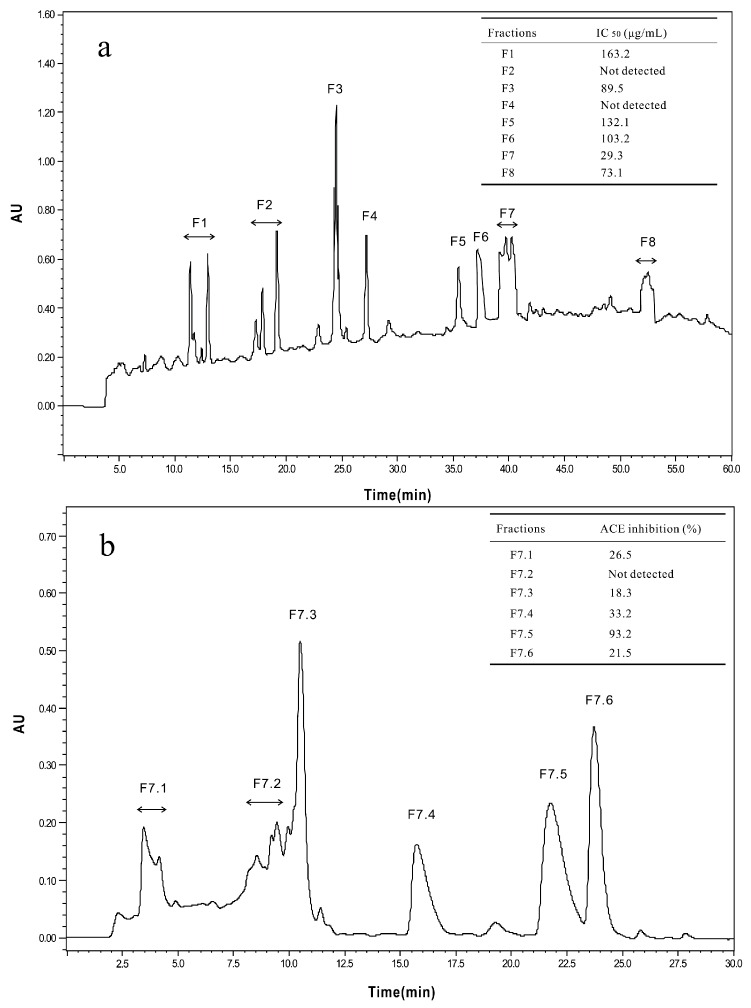
Chromatograms of RP-HPLC for the two-step method used to purify and assay the ACE-inhibitory peptides. (**a**) First step of RP-HPLC for fraction G5 from the Sephadex G-15 gel filtration; (**b**) Second step of RP-HPLC for fraction F7, the ACE-inhibitory activities of factions (F7.1–F7.6) were determined at a concentration of 30 μg/mL.

The <3 kDa fraction of *A. elegans* T3 proteases hydrolysate was separated by Sephadex G-15 gel filtration chromatography into five major absorbance peaks at 220 nm (Figure 3). Fractions (G1–G5) associated with the peaks were pooled and lyophilized for ACE-inhibitory activity assay. The fraction G5 exhibited the highest ACE-inhibitory activity among the collected fractions, with IC_50_ value of 0.17 mg/mL. Therefore, the fraction G5 was subjected to RP-HPLC for further purification. Eight peaks (F1–F8) were obtained separately according to the chromatogram (60 min) (Figure 4a). The highest inhibitory activity was observed in fraction F7, with an IC_50_ value of 29.3 μg/mL. Fraction F7 was further purified by the second step of RP-HPLC and fractionated into six major sub-fractions (F7.1–F7.6, Figure 4b). Most of the ACE-inhibitory activity occurred in fraction F7.5, which inhibited 96.2% of the ACE activity at the concentration of 30 μg/mL, whereas the inhibitory activities of the other sub-fractions were below 35%. Thereafter, fraction F7.5 was selected to identify its sequence by MALDI/TOF-TOF MS/MS.

#### 2.2.2. Determination of Amino Acids Sequence

**Figure 5:**
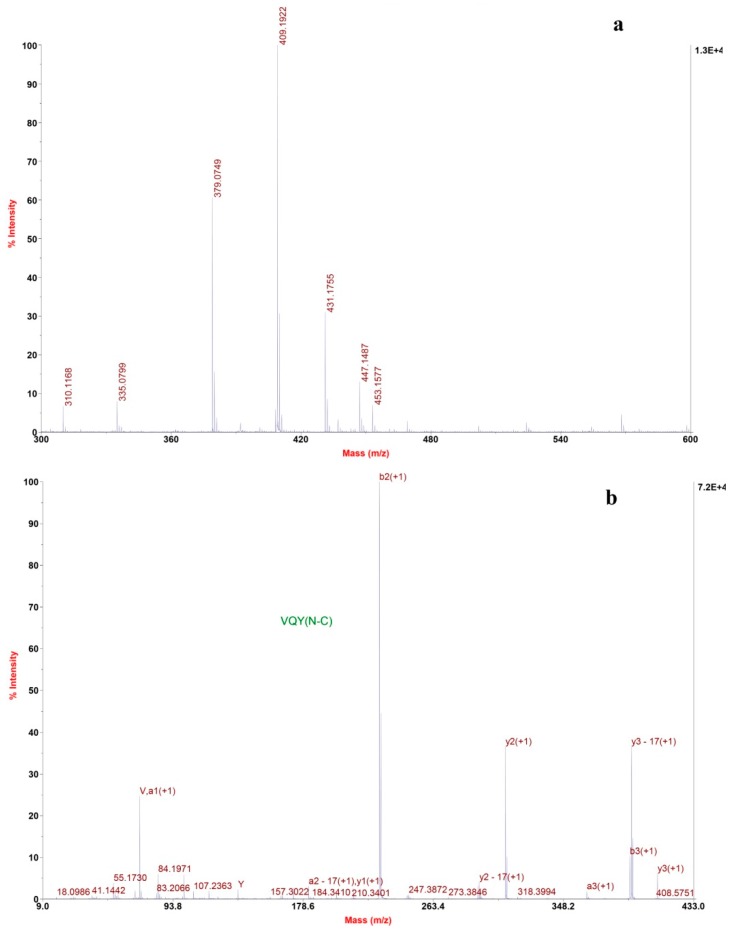
*De novo* sequencing of purified ACE-inhibitory peptide from RP-HPLC. (**a**) MALDI/TOF-TOF MS spectrum of the purified peptide; (**b**) MALDI/TOF-TOF MS/MS spectrum of the ion 409.2 *m/z*.

The mass spectrum of fraction F7.5 revealed one most intensive signal, indicating a single positively charged ion ([M + H]^+^) at 409.2 (Figure 5a). Several other signals with moderate intensity were seen on the spectrum. Tandem mass spectra confirmed that they are not peptides. The molecular mass of fraction F7.5 was determined to be 408.2 Da, and ion at *m/z* 409.2 was selected as precursor ion for TOF-TOF tandem MS analysis. The amino acid sequence was obtained by de novo sequencing using software from the MS/MS spectrum (Figure 5b). Also, the masses of the singly charged ions were matched to the single peptide fragment by manual validation. Therefore, the amino sequence of fraction F7.5 was identified as Val-Gln-Tyr.

#### 2.2.3. IC_50_ Value and Inhibition Pattern of Val-Gln-Tyr

**Figure 6:**
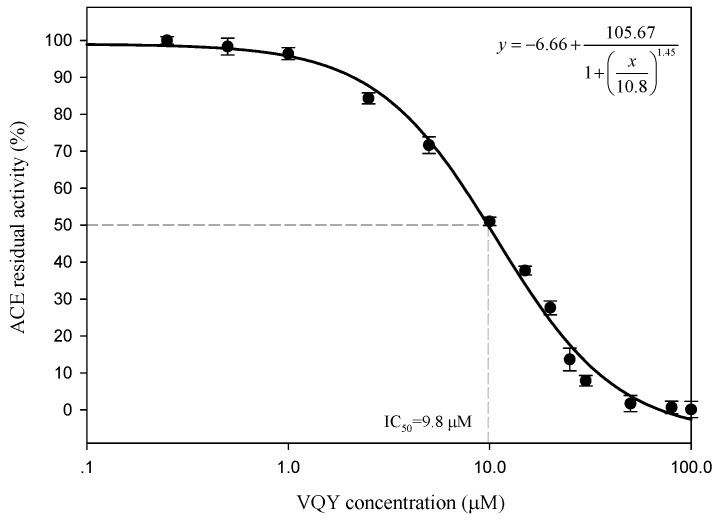
Determination of IC_50_ value of VQY.

**Figure 7:**
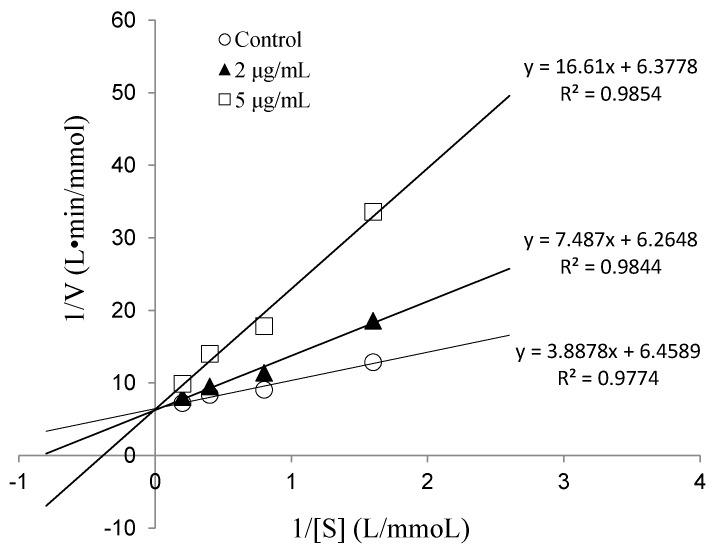
Lineweaver-Burk plots of VQY inhibition on ACE.

To determine the IC_50_ value and ACE inhibition pattern, Val-Gln-Tyr (VQY) was chemically synthesized with a purity of greater than 98% by solid-phase technique (Chinese peptide Co., Ltd., Hangzhou, China). The IC_50_ value of VQY was estimated by non-linear regression by fitting the results of ACE-inhibitory activity (assayed at different concentrations of inhibitor, 0.25–100 μM) to a four-parameter logistic equation (Figure 6). The nonlinear regression coefficient of the equation (*R* = 0.977) demonstrates that the actual value of the experimental data corresponds well with the value predicted by the equation. The IC_50_ value of VQY was determined as 9.8 μM by solving the equation. Many potent ACE-inhibitory peptides have been isolated and identified from various food proteins. Among them, IPP and VPP are well characterized ACE-inhibitory peptides from fermented milk with IC_50_ values of 5 μM and 9 μM, respectively. The IC_50_ value of VQY reported in this study is close to these two peptides and another peptide VLP isolated from freshwater clam (*Corbicula fluminea*) with an IC_50_ value of 3.7 μM [37]. However, the IC_50_ value of VQY peptide reported in this study is much lower than YN peptide (51 μM) isolated from the hard clam *Meretrix lusoria* [14]. To the best of our knowledge, this peptide (VQY) is a novel peptide derived from razor clam proteins exhibiting a strong ACE-inhibitory activity. Structure-activity correlation among ACE-inhibitory peptides shows that their activity is strongly influenced by amino acid residues of peptide sequence [38,39]. Many studies have shown that potential ACE-inhibitory peptides exhibit hydrophobic amino acid residues (tryptophan, phenylalanine, tyrosine, or proline) at their *C*-terminus while contain branched aliphatic amino acid residues (Val, Ile, Leu) at the *N*-terminus [40,41]. The peptide VQY is in accordance with this rule, containing valine at the *N*-terminal and tyrosine at the *C*-terminal. Lineweaver-Burk plots of VQY for ACE inhibition showed three lines, representing ACE reaction performed in the absence and presence of the peptide. The lines intersected at one point on the vertical axis, which indicates a competitive inhibition pattern (Figure 7). This result suggests that the peptide (VQY) acts as a competitive inhibitor and razor clam hydrolysate is a potential candidate of antihypertensive nutraceuticals.

## 3. Materials and Methods

### 3.1. Materials

Samples of razor clams (*Sinonovacula constricta*) were obtained from local market. *Actinomucor elegans* T3 with strong proteolytic activity was isolated from a traditional fermented soybean product. ACE (EC 3.4.15.1, from rabbit lung), Hippurl-1-histidyl-l-leucine (HHL), Pepsin (P6887) and Trypsin (T1426) were purchased from Sigma-Aldrich (St. Louis, MO, USA). Alcalase 2.4 L and Flavourzyme 500 MG were purchased from Novozyme (Bagasvaerd, Denmark). All other chemicals were also of analytical grade.

### 3.2. Preparation of Crude Proteases from Actinomucor elegans T3

Production of crude enzyme from *Actinomucor elegans* T3 was obtained according to the following method. *A. elegans* T3 was grown on Potato Dextrose Agar (PDA) at 28 °C for 72 h. Firstly, the inoculum was prepared by transferring three round blocks (6 mm in diameter), cut from the plate culture, into 100 mL PDB (Potato Dextrose Broth). The culture was allowed to grow at 28 °C for 2 days on a shaking incubator at 150 rpm. Twenty milliliters of the inoculum was then transferred into 500 mL flasks containing 180 mL of medium for proteases production. The composition of the medium was as given (L^−1^): 15 g glucose, 10 g soy protein isolate, 2.5 g yeast extract, 2 g KH_2_PO_4_, 2 g MgSO_4_ with a final pH of 6.0. After inoculation, the medium containing the culture was incubated at 28 °C on a shaking incubator upheld at 150 rpm for 60 h. The supernatants were collected by centrifugation (10,000× *g*, 15 min) at 4 °C and then passed through 0.45 μm filters. The filtrates were lyophilized and used as crude proteases. The lyophilized filtrates were stored at −20 °C until use. One unit of proteases was defined as the amount of enzyme required to bring an increase of 0.01 OD units at 280 nm per minute at assay conditions and measured as 0.4 M Trichloroacetic acid (TCA) soluble products using hemoglobin as substrate.

### 3.3. Enzymatic Hydrolysis

Meat of razor clams was stripped from the shell completely and washed carefully with distilled water to remove sand. Clean tissues were homogenized with distilled water (two times the volume of the tissues). The homogenate was heated at 85 °C for 10 min to inactivate endogenous proteases and then lyophilized. The resulting razor clam powder was kept at −20 °C until hydrolysis. Proximate composition of razor clam was determined according to the method of the Association of Official Analytical Chemists [42].

For hydrolysis with each protease, twenty grams of razor clam powder was mixed with 200 mL of distilled water in a blender for 2 min. Protease was added to the mixture at the enzyme/substrate ratio of 3000 U/g. The hydrolysis reactions were conducted under optimal conditions of different proteases (Table 2). During the hydrolysis, the pH value was kept at the optimal level by adding 1 M HCL or 1 M NaOH. The reaction was stopped by heating the mixture at 90 °C for 10 min followed by centrifugation at 8000× *g* for 20 min at 4 °C. Samples from the supernatants were subjected to peptide content assay. The other collected supernatants were ultra-filtrated sequentially through 3 and 10 kDa molecular weight cutoff membranes (MWCO) (Millipore). The supernatants were first passed through the membranes with MWCO of 10 kDa. The retentate from 10 kDa membrane was collected and designated as >10 kDa fraction. The permeate solution collected from 10 kDa membrane was then filtered through the membrane with MWCO of 3 kDa. Retentate and permeate samples collected from 3 kDa membrane were designated as 3–10 kDa and <3 kDa fractions, respectively. All these collected fractions were then lyophilized and stored at −20 °C until further analysis.

### 3.4. Analytical Methods

#### 3.4.1. Angiotensin-Converting Enzyme Inhibition Assay

The ACE-inhibitory activity was measured by HPLC according to the method described by Cushman and Cheung [43] using HHL as a substrate. The total volume of ACE reaction system was 100 uL consisting of the following components: 50 μL substrate solution (5 mM HHL in 50 mM HEPES with 300 mM NaCl, pH 8.3), 40 μL test sample and 10 μL ACE (0.1 U/mL). The substrate solution and sample were mixed and incubated at 37 °C for 5 min in a water bath. Then ACE was added and incubated at 37 °C for 30 min. The reaction was terminated by adding 250 μL of 1 M HCl. Hippuric acid (HA) released from ACE reaction was measured by RP-HPLC (Agilent Inc., Santa Clara, CA, USA) equipped with C18 column (4.6 × 150 mm, 5 μm, Thermo Scientific, Waltham, MA, USA) and absorbance detector set at 228 nm. The HHL and HA were eluted using a gradient of 21% (*v*/*v*) acetonitrile containing 0.5% (*v*/*v*) trifluoroacetic acid at a flow rate of 1 mL/min. The inhibitory activity was calculated using the following formula:

(1) $$I\left(\%\right)=\frac{A-B}{A}\times 100$$

where *I* is the percentage of ACE inhibition by sample, *A* is the concentration of HA of blank test by using distilled water instead of sample and *B* is the concentration of HA with sample added. The IC_50_ value was defined as the concentration of peptide inhibiting 50% of the ACE activity under the assayed conditions, which was estimated by non-linear regression by fitting data to a four-parameter logistic curve using SigmaPlot software (version 10.0, SPSS Inc., Chicago, IL, USA).

#### 3.4.2. Degree of Hydrolysis Evaluation

Degree of hydrolysis (DH) was estimated by measuring the content of α-amino groups released by hydrolysis according to the *o*-phthaldialdehyde (OPA) method [44]. The content of α-amino groups was expressed as the concentration of serine corresponding to standard curve. The DH was calculated using the following equation.

(2) $$DH\left(\%\right)=\frac{B-A}{C-A}\times 100$$

*A* is the content of α-amino group at the beginning of protease hydrolysis, and *B* is the content of α-amino group in the supernatant after hydrolysis. *C* is the content of α-amino group from the razor clam powder hydrolyzed with 6 M HCl (containing 1% (*v*/*v*) phenol) at 110 °C for 12 h in tubes sealed under nitrogen.

#### 3.4.3. Determination of Peptide Content

The peptide content was determined by the Folin phenol method [45] using synthetic peptide Tyr-Gly-Gly-Phe-Leu-Arg-Lys-Tyr (with molecular weight of 1003.17 g/mol, Chinese peptide Co. Ltd., Hangzhou, China) as standard.

### 3.5. Purification and Identification of ACE-Inhibitory Peptides

#### 3.5.1. Gel Filtration Chromatography

The lyophilized powder of ultra-filtration permeate was dissolved in distilled water at a concentration of 100 mg/mL. Two milliliter of the solution was loaded onto a Sephadex G-15 column (1.8 × 60 cm) eluted with distilled water at a flow rate of 0.5 mL/min. Fractions were collected at 5 min intervals and the absorbance was measured at 220 nm. The active fractions were pooled and lyophilized for further purification.

#### 3.5.2. Reversed-Phase High-Performance Liquid Chromatography

The selected fraction obtained from gel filtration was re-dissolved in ultrapure water at a concentration of 10 mg/mL. Five hundred microliters was injected into Waters 600 HPLC system (semi-preparative RP-HPLC, Waters, Milford, MA, USA) equipped with Kromasil C18 column (10 × 250 mm, 10 μm). Solvent A was 0.1% (*v/v*) trifluoroacetic acid (TFA) in ultrapure water and solvent B was 0.1% (*v/v*) TFA in 80% (*v/v*) acetonitrile. The elution was 100% solvent A for 5 min, followed by a linear gradient from 0% to 55% of solvent B in 60 min at a flow rate of 2 mL/min. The absorbance of eluent was detected with a UV detector at 220 nm. Fractions were collected separately through repeated chromatography using RP-HPLC and concentrated for ACE-inhibitory activity assay. The fraction with the highest inhibitory activity was lyophilized and dissolved at 5 mg/mL concentration for the second step RP-HPLC separation under the similar conditions. Two hundred microliters of the samples was injected and further separated at a flow rate of 1 mL/min with a linear gradient elution of 25%–40% solvent B for 30 min. The peak with the most of the inhibitory activity was collected and lyophilized.

#### 3.5.3. Identification of the Amino Acid Sequence by MALDI/TOF-TOF MS/MS

The amino acid sequence of the purified peptide was identified by MALDI–TOF–MS/MS. Peptide sample (0.5 μL) was mixed with 0.5 μL of a saturated solution of α-cyano-4-hydroxycinnamic acid in 50% (*v/v*) acetonitrile containing 0.1% (*v/v*) TFA. The mixture was spotted on the target plate and analyzed in ABI 5700 MALDI-TOF/TOF MS/MS (AB Sciex, Framingham, MA, USA) in positive reflector mode with a mass range from 300 to 1000 *m/z*. The amino acid sequence of peptide fragments was determined by de novo sequencing using the software DeNovo Explorer (version4.5, AB Sciex, Framingham, MA, USA) and confirmed by manual validation.

#### 3.5.4. Determination of ACE-Inhibition Pattern

The inhibition kinetics of the peptide on ACE was investigated using HHL as a substrate. Lineweaver-Burk plot was used to determine the type of inhibition of the peptide. The ACE reactions were carried out at various substrate concentrations (0.625, 1.25, 2.5 and 5 mM) in the absence and presence of two different concentrations of the peptide (2 and 5 μg/mL). Linear interpolation was plotted with the reciprocal of HHL concentration (1/[*S*]) as the independent variable and with the reciprocal of HA production (1/[*V*]) as the dependent variable [46].

### 3.6. Statistical Analysis

The results were expressed as mean ±SD (standard deviation). The statistics analysis was carried out using SPSS 20.0 (version 20, SPSS Inc., Chicago, IL, USA). Differences among treatments were determined by one way ANOVA. The *p* value less than 0.05 was considered as statistically significant.

## 4. Conclusions

The present study revealed that enzyme hydrolysates of razor clam have good potential for the production of ACE-inhibitory peptides. Among the proteases tested in this trial, *A. elegans* T3 proteases was found to be the most efficient in producing small peptides with the best ACE-inhibitory activity. A novel potent ACE-inhibitory peptide, VQY, with the IC_50_ value of 9.8 μM, was purified from the hydrolysate by a series of chromatographic separations and identified by MALDI/TOF-TOF MS/MS. Lineweaver-Burk plots revealed that the peptide exhibits strong competitive inhibition activity against ACE. This is the first report of ACE-inhibitory peptides derived from enzymatic hydrolysates of razor clam. It is highly recommended that the ACE-inhibitory peptides from razor clam hydrolysates be employed in the development of nutraceuticals and pharmaceuticals for the treatment of hypertension.

## Figures and Tables

**Table 1:** Proximate composition of razor clam.

Composition	Contents (g/100 g Fresh Weight)
Moisture	80.32 ± 0.53
Protein	13.68 ± 0.62
Fat	1.89 ± 0.13
Carbohydrate	2.13 ± 0.31
Ash	1.93 ± 0.08

**Table 2:** Hydrolysis conditions of proteases.

Protease	Source	Temperature (°C)	pH
Pepsin	porcine gastric mucosa	37	2.0
Trypsin	bovine pancreas	37	8.0
Alcalase	*Bacillus licheniformis*	40	8.0
Flavourzyme	*Aspergillus oryzae*	50	6.0
Crude proteases	*Actinomucor elegans*	55	6.0

## References

[1] Poulter N.R., Prabhakaran D., Caulfield M. (2015). Hypertension. Lancet.

[2] Lackland D.T., Weber M.A. (2015). Global burden of cardiovascular disease and stroke: Hypertension at the core. Can. J. Cardiol..

[3] George B., Pantelis S., Rajiv A., Luis R. (2014). Review of blood pressure control rates and outcomes. J. Am. Soc. Hypertens..

[4] FerrãO F.M., Lara L.S., Lowe J. (2014). Renin-angiotensin system in the kidney: What is new?. World J. Nephrol..

[5] Cheung R.C., Ng T.B., Wong J.H. (2015). Marine peptides: Bioactivities and applications. Mar. Drugs.

[6] Wu R., Wu C., Liu D., Yang X., Huang J., Zhang J., Liao B., He H., Li H. (2015). Overview of antioxidant peptides derived from marine resources: The sources, characteristic, purification, and evaluation methods. Appl. Biochem. Biotechnol..

[7] Fan X., Bai L., Zhu L., Yang L., Zhang X. (2014). Marine algae-derived bioactive peptides for human nutrition and health. J. Agric. Food Chem..

[8] Bartneck M., Heffels K.H., Pan Y., Bovi M., Zwadlo-Klarwasser G., Groll J. (2012). Inducing healing-like human primary macrophage phenotypes by 3D hydrogel coated nanofibres. Biomaterials.

[9] Ferreira S.H., Bartelt D.C., Greene L.J. (1970). Isolation of bradykinin-potentiating peptides from *Bothrops jararaca* venom. Biochemistry.

[10] Saadi S., Saari N., Anwar F., Abdul Hamid A., Ghazali H.M. (2015). Recent advances in food biopeptides: Production, biological functionalities and therapeutic applications. Biotechnol. Adv..

[11] Shiozaki K., Shiozaki M., Masuda J., Yamauchi A., Ohwada S., Nakano T., Yamaguchi T., Saito T., Muramoto K., Sato M. (2010). Identification of oyster-derived hypotensive peptide acting as angiotensin-I-converting enzyme inhibitor. Fish. Sci..

[12] Wang J., Hu J., Cui J., Bai X., Du Y., Miyaguchi Y., Lin B. (2008). Purification and identification of a ACE inhibitory peptide from oyster proteins hydrolysate and the antihypertensive effect of hydrolysate in spontaneously hypertensive rats. Food Chem..

[13] He H.L., Chen X.L., Sun C.Y., Zhang Y.Z., Zhou B.C. (2006). Analysis of novel angiotensin-I-converting enzyme inhibitory peptides from protease-hydrolyzed marine shrimp *Acetes chinensis*. J. Pept. Sci..

[14] Tsai J.S., Pan C.B.S. (2008). ACE-inhibitory peptides identified from the muscle protein hydrolysate of hard clam (*Meretrix lusoria*). Process Biochem..

[15] Balti R., Nedjar-Arroume N., Adje E.Y., Guillochon D., Nasri M. (2010). Analysis of novel angiotensin I-converting enzyme inhibitory peptides from enzymatic hydrolysates of cuttlefish (*Sepia officinalis*) muscle proteins. J. Agric. Food Chem..

[16] Feng B., Dong L., Niu D., Meng S., Zhang B., Liu D., Hu S., Li J. (2010). Identification of immune genes of the *Agamaki clam* (*Sinonovacula constricta*) by sequencing and bioinformatic analysis of ests. Mar. Biotechnol..

[17] Karnjanapratum S., Benjakul S., Kishimura H., Tsai Y.H. (2013). Chemical compositions and nutritional value of Asian hard clam (*Meretrix lusoria*) from the coast of Andaman Sea. Food Chem..

[18] Yoon H., An Y.K., Choi S.D., Kim J. (2008). Proximate composition in the muscle and viscera of five Veneridae clams (bivalvia) from southern coast of Korea. Korean J. Matacol..

[19] Laxmilatha P. (2009). Proximate composition of the surf clam *Mactra violacea* (Gmelin 1791). Indian J. Fish..

[20] Bougatef A., Nedjar-Arroume N., Ravallec-Plé R., Leroy Y., Guillochon D., Barkia A., Nasri M. (2008). Angiotensin I-converting enzyme (ACE) inhibitory activities of sardinelle (*Sardinella aurita*) by-products protein hydrolysates obtained by treatment with microbial and visceral fish serine proteases. Food Chem..

[21] Giménez B., Alemán A., Montero P., Gómez-Guillén M. (2009). Antioxidant and functional properties of gelatin hydrolysates obtained from skin of sole and squid. Food Chem..

[22] Klompong V., Benjakul S., Kantachote D., Shahidi F. (2007). Antioxidative activity and functional properties of protein hydrolysate of yellow stripe trevally (*Selaroides leptolepis*) as influenced by the degree of hydrolysis and enzyme type. Food Chem..

[23] Mahmoodani F., Ghassem M., Babji A.S., Yusop S.M., Khosrokhavar R. (2014). ACE inhibitory activity of pangasius catfish (*Pangasius sutchi*) skin and bone gelatin hydrolysate. J. Food Sci. Technol..

[24] Benjakul S., Morrissey M.T. (1997). Protein hydrolysates from pacific whiting solid wastes. J. Agric. Food Chem..

[25] Nguyen H.T.M., Sylla K.S.B., Randriamahatody Z., Donnay-Moreno C., Moreau J., Tran L.T., Bergé J.P. (2011). Enzymatic hydrolysis of yellowfin tuna (*Thunnus albacares*) by-products using protamex protease. Food Technol. Biotechnol..

[26] Guerard F., Guimas L., Binet A. (2002). Production of tuna waste hydrolysates by a commercial neutral protease preparation. J. Mol. Catal. B Enzym..

[27] Hausch F., Shan L., Santiago N.A., Gray G.M., Khosla C. (2002). Intestinal digestive resistance of immunodominant gliadin peptides. Am. J. Physiol. Gastrointest. Liver Physiol..

[28] Bamdad F., Wu J.P., Chen L.Y. (2011). Effects of enzymatic hydrolysis on molecular structure and antioxidant activity of barley hordein. J. Cereal Sci..

[29] Adler-Nissen J. (1982). Limited enzymic degradation of proteins: A new approach in the industrial application of hydrolases. J. Chem. Technol. Biotechnol..

[30] Wu J.P., Aluko R.E., Muir A.D. (2009). Production of angiotensin I-converting enzyme inhibitory peptides from defatted canola meal. Bioresour. Technol..

[31] Zarei M., Forghani B., Ebrahimpour A., Abdul-Hamid A., Anwar F., Saari N. (2015). *In vitro* and *in vivo* antihypertensive activity of palm kernel cake protein hydrolysates: Sequencing and characterization of potent bioactive peptides. Ind. Crop. Prod..

[32] Zhang Y., Olsen K., Grossi A., Otte J. (2013). Effect of pretreatment on enzymatic hydrolysis of bovine collagen and formation of ACE-inhibitory peptides. Food Chem..

[33] Cheung I.W.Y., Li-Chan E.C.Y. (2014). Application of taste sensing system for characterisation of enzymatic hydrolysates from shrimp processing by-products. Food Chem..

[34] Aissaoui N., Abidi F., Marzouki M.N. (2015). ACE inhibitory and antioxidant activities of red scorpionfish (*Scorpaena notata*) protein hydrolysates. J. Food Sci. Technol. Mysore.

[35] Hernandez-Ledesma B., Contreras M.D., Recio I. (2011). Antihypertensive peptides: Production, bioavailability and incorporation into foods. Adv. Colloid Interface Sci..

[36] Ramanathan N., Schwager S.L.U., Sturrock E.D., Acharya K.R. (2003). Crystal structure of the human angiotensin-converting enzyme-lisinopril complex. Nature.

[37] Tsai J.S., Lin T.C., Chen J.L., Pan B.S. (2006). The inhibitory effects of freshwater clam (*Corbicula fluminea*, Muller) muscle protein hydrolysates on angiotensin I converting enzyme. Process Biochem..

[38] Cristina M., Maria D.M.Y., Justo P., Hassan L., Julio G.C., Manuel A., Francisco M., Javier V. (2004). Purification of an ACE inhibitory peptide after hydrolysis of sunflower (*Helianthus annuus* L.) protein isolates. J. Agric. Food Chem..

[39] Wu J., Ding X. (2001). Hypotensive and physiological effect of angiotensin converting enzyme inhibitory peptides derived from soy protein on spontaneously hypertensive rats. J. Agric. Food Chem..

[40] Wu J., Aluko R.E., Shuryo N. (2006). Structural requirements of angiotensin I-converting enzyme inhibitory peptides: Quantitative structure-activity relationship study of di- and tripeptides. J. Agric. Food Chem..

[41] Rohrbach M.S., Williams E.B., Rolstad R.A. (1981). Purification and substrate specificity of bovine angiotensin-converting enzyme. J. Biol. Chem..

[42] Helrich K. (1990). Official Methods of Analysis of the AOAC.

[43] Cushman D.W., Cheung H.S. (1971). Spectrophotometric assay and properties of the angiotensin I-converting enzyme of rabbit lung. Biochem. Pharmacol..

[44] Nielsen P.M., Petersen D., Dambmann C. (2001). Improved method for determining food protein degree of hydrolysis. J. Food Sci..

[45] Lowry O.H., Rosebrough N.J., Farr A.L., Randall R.J. (1951). Protein measurement with the Folin phenol reagent. J. Biol. Chem..

[46] Rao S.Q., Ju T., Sun J., Su Y.J., Xu R.R., Yang Y.J. (2012). Purification and characterization of angiotensin I-converting enzyme inhibitory peptides from enzymatic hydrolysate of hen egg white lysozyme. Food Res. Int..

